# Sparse signals recovered by non-convex penalty in quasi-linear systems

**DOI:** 10.1186/s13660-018-1652-8

**Published:** 2018-03-14

**Authors:** Angang Cui, Haiyang Li, Meng Wen, Jigen Peng

**Affiliations:** 1 School of Mathematics and Statistics, Xi’an Jiaotong University, Xi’an, China; 2 School of Science, Xi’an Polytechnic University, Xi’an, China

**Keywords:** 34A34, 78M50, 93C10, Compressed sensing, Quasi-linear, Non-convex fraction function, Iterative thresholding algorithm

## Abstract

The goal of compressed sensing is to reconstruct a sparse signal under a few linear measurements far less than the dimension of the ambient space of the signal. However, many real-life applications in physics and biomedical sciences carry some strongly nonlinear structures, and the linear model is no longer suitable. Compared with the compressed sensing under the linear circumstance, this nonlinear compressed sensing is much more difficult, in fact also NP-hard, combinatorial problem, because of the discrete and discontinuous nature of the $\ell _{0}$-norm and the nonlinearity. In order to get a convenience for sparse signal recovery, we set the nonlinear models have a smooth quasi-linear nature in this paper, and study a non-convex fraction function $\rho_{a}$ in this quasi-linear compressed sensing. We propose an iterative fraction thresholding algorithm to solve the regularization problem $(QP_{a}^{\lambda})$ for all $a>0$. With the change of parameter $a>0$, our algorithm could get a promising result, which is one of the advantages for our algorithm compared with some state-of-art algorithms. Numerical experiments show that our method performs much better than some state-of-the-art methods.

## Introduction

In compressed sensing (see, e.g., [1, 2]), the problem of reconstructing a sparse signal under a few linear measurements which are far fewer than the dimension of the ambient space of the signal can be modeled into the following $\ell _{0}$-minimization:

1 $$ (P_{0}) \quad \min_{x\in\mathbb{R}^{n}} \Vert x \Vert _{0} \quad\text{subject to } Ax=b, $$

where $A\in\mathbb{R}^{m\times n}$ is an $m\times n$ real matrix of full row rank with $m< n$, and $b\in\mathbb{R}^{m}$ is a nonzero real vector of *m*-dimension, and $\Vert x \Vert _{0}$ is the $\ell_{0}$-norm of the real vector *x*, which counts the number of the nonzero entries in *x* (see, e.g., [3–5]). In general, the problem $(P_{0})$ is computational and NP-hard because of the discrete and discontinuous nature of the $\ell_{0}$-norm. However, many real-life applications in physics and biomedical sciences carry some strongly nonlinear structures [6], so that the linear model in problem $(P_{0})$ is no longer suitable. In this nonlinear case, we consider a map $A: \mathbb{R}^{n}\rightarrow\mathbb{R}^{m}$, which is no longer necessarily linear, and reconstruct a sparse vector $x\in \mathbb{R}^{n}$ from the measurements $b\in\mathbb{R}^{m}$ given by

2 $$ A(x)=b. $$

Compared with the $\ell_{0}$-minimization under the linear circumstance, this nonlinear minimization is much more difficult, in fact also NP-hard, combinatorial problem, because of the discrete and discontinuous nature of the $\ell_{0}$-norm and the nonlinearity. In order to get a convenience for sparse signal recovery, in this paper, we set the nonlinear models have a smooth quasi-linear nature. By this means, there exists a Lipschitz map

3 $$ F: \mathbb{R}^{n}\rightarrow\mathbb{R}^{m\times n} $$

such that

4 $$ A(x)=F(x)x $$

for all $x\in\mathbb{R}^{n}$. So, the $\ell_{0}$-minimization under the quasi-linear case can be mathematically viewed as the following form:

5 $$ (QP_{0})\quad \min_{x\in\mathbb{R}^{n}} \Vert x \Vert _{0}\quad \text{subject to } F(x)x=b. $$

In fact, the minimization ($QP_{0}$) under the quasi-linear case is also combinatorial and NP-hard [6, 7]. To overcome this problem, the authors in [6, 7] proposed the $\ell _{1}$-minimization

6 $$ (QP_{1}) \quad \min_{x\in\mathbb{R}^{n}} \Vert x \Vert _{1} \quad\text{subject to } F(x)x=b $$

for the constrained problem and

7 $$ \bigl(QP_{1}^{\lambda}\bigr)\quad \min _{x\in\mathbb{R}^{n}} \bigl\{ \bigl\Vert F(x)x-b \bigr\Vert _{2}^{2}+\lambda \Vert x \Vert _{1} \bigr\} $$

for the regularization problem, where $\Vert x \Vert _{1}=\sum_{i=1}^{n} \vert x_{i} \vert $ is the $\ell_{1}$-norm of vector *x*.

In [6, 7], the authors have shown that the $\ell _{1}$-norm minimization can really make an exact recovery in some specific conditions. In general, however, these conditions are always hard to satisfied in practice. Moreover, the regularization problem $(QP_{1}^{\lambda})$ always leads to a biased estimation by shrinking all the components of the vector toward zero simultaneously, and sometimes results in over-penalization in the regularization model $(QP_{1}^{\lambda})$ as the $\ell_{1}$-norm in linear compressed sensing.

In pursuit of better reconstruction results, in this paper, we propose the following fraction minimization:

8 $$ \bigl(QP_{a}^{\lambda}\bigr)\quad \min _{x\in\mathbb{R}^{n}} \bigl\{ \bigl\Vert F(x)x-b \bigr\Vert _{2}^{2}+\lambda P_{a}(x) \bigr\} , $$

where

9 $$ P_{a}(x)=\sum_{i=1}^{n} \rho_{a}(x_{i}),\quad a>0 $$

and

10 $$ \rho_{a}(t)=\frac{a \vert t \vert }{a \vert t \vert +1} $$

is the fraction function which performs outstanding in image restoration [8], linear compressed sensing [9] and matrix rank minimization problem [10]. Clearly, with the change of parameter $a>0$, the non-convex function $P_{a}(x)$ could approximately interpolate the $\ell_{0}$-norm

11 $$ \lim_{a\rightarrow+\infty}\rho_{a}(x_{i})= \textstyle\begin{cases} 0 & \text{if } x_{i}=0; \\ 1 & \text{if } x_{i}\neq0. \end{cases} $$

**Figure 1:**
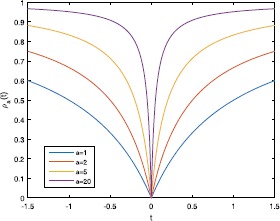
Behavior of the fraction function $\rho_{a}(t)$ for various values of $a>0$

Figure 1 shows the behavior of the fraction function $\rho_{a}(t)$ for various values of $a>0$.

The rest of this paper is organized as follows. Some preliminary results that are used in this paper are given in Sect. 2. In Sect. 3, we propose an iterative fraction thresholding algorithm to solve the regularization problem $(QP_{a}^{\lambda})$ for all $a>0$. In Sect. 3, we present some numerical experiments to demonstrate the effectiveness of our algorithm. The concluding remarks are presented in Sect. 4.

## Preliminaries

In this section, we give some preliminary results that are used in this paper.

Define a function of $\beta\in\mathbb{R}$ as

12 $$ f_{\lambda}(\beta)=(\beta-\gamma)^{2}+\lambda \rho_{a}(\beta) $$

and let

13 $$ \mathrm{prox}_{a,\lambda}^{\beta}(\gamma)\triangleq\arg \min_{\beta\in\mathbb{R}}f_{\lambda}(\beta). $$

### Lemma 1

(see [9–11])

*The operator* $\mathrm{prox}_{a,\lambda}^{\beta}$ *defined in* (13) *can be expressed as*

14 $$ \mathrm{prox}_{a,\lambda}^{\beta}(\gamma)= \textstyle\begin{cases} g_{a,\lambda}(\gamma) & \textit{if } { \vert \gamma \vert > t_{a,\lambda}^{\ast};} \\ 0& \textit{if } { \vert \gamma \vert \leq t_{a,\lambda}^{\ast},} \end{cases} $$

*where* $g_{a,\lambda}(\gamma)$ *is defined as*

15 $$\begin{aligned} & g_{a,\lambda}(\gamma)=\operatorname{sign}(\gamma) \biggl(\frac{\frac{1+a \vert \gamma \vert }{3}(1+2\cos(\frac{\phi(\gamma)}{3}-\frac{\pi}{3}))-1}{a} \biggr), \\ &\phi(\gamma)=\arccos\biggl(\frac{27\lambda a^{2}}{4(1+a \vert \gamma \vert )^{3}}-1\biggr) \end{aligned}$$

*and the threshold value satisfies*

16 $$ t_{a,\lambda}^{\ast}=\textstyle\begin{cases} t_{a,\lambda}^{1} & \textit{if } {\lambda\leq\frac {1}{a^{2}};} \\ t_{a,\lambda}^{2} & \textit{if } {\lambda>\frac{1}{a^{2}},} \end{cases} $$

*where*

17 $$ t_{a,\lambda}^{1}=\frac{\lambda}{2}a,\qquad t_{a,\lambda }^{2}=\sqrt{\lambda}-\frac{1}{2a}. $$

**Figure 2:**
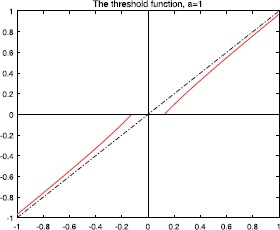
Plot of the threshold function $g_{a,\lambda}$ for $a=1$ and $\lambda=0.25$

**Figure 3:**
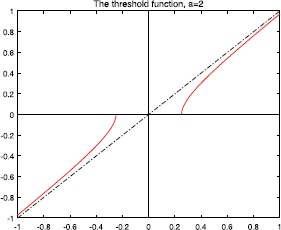
Plot of the threshold function $g_{a,\lambda}$ for $a= 2$ and $\lambda=0.25$

**Figure 4:**
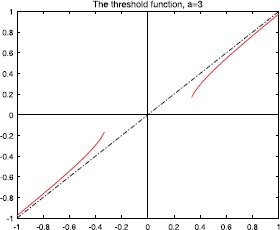
Plot of the threshold function $g_{a,\lambda}$ for $a= 3$ and $\lambda=0.25$

**Figure 5:**
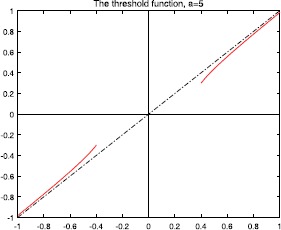
Plot of the threshold function $g_{a,\lambda}$ for $a=5$, and $\lambda=0.25$

Figures 2, 3, 4, and 5 show the plots of the threshold function $g_{a,\lambda}$ for $a=1, 2, 3, 5$, and $\lambda=0.25$.

**Figure 6:**
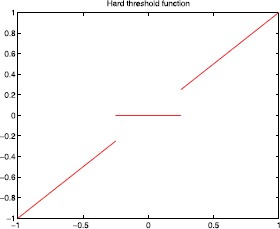
Plot of the hard threshold function with $\lambda =0.25$

**Figure 7:**
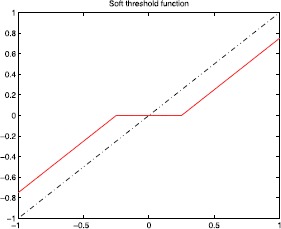
Plot of the soft threshold function with $\lambda =0.25$

Figures 6 and 7 show the plots of the hard/soft threshold functions with $\lambda=0.25$.

### Definition 1

The iterative thresholding operator $G_{a, \lambda}$ can be defined by

18 $$ G_{a, \lambda}(x)=\bigl(\mathrm{prox}_{a,\lambda}^{\beta}(x_{1}), \ldots, \mathrm{prox}_{a,\lambda}^{\beta}(x_{n}) \bigr)^{\top}, $$

where $\mathrm{prox}_{a,\lambda}^{\beta}$ is defined in Lemma 1.

## Thresholding representation theory and algorithm for problem $(QP_{a}^{\lambda})$

In this section, we establish a thresholding representation theory of the problem $(QP_{a}^{\lambda})$, which underlies the algorithm to be proposed. Then an iterative fraction thresholding algorithm (IFTA) is proposed to solve the problem $(QP_{a}^{\lambda})$ for all $a>0$.

### Thresholding representation theory

For any fixed positive parameters $\lambda>0$, $\mu>0$, $a>0$ and $x\in\mathbb{R}^{n}$, let

19 $$ C_{1}(x)= \bigl\Vert F(x)x-b \bigr\Vert _{2}^{2}+\lambda P_{a}(x) $$

and

20 $$ C_{2}(x, y)=\mu \bigl\Vert F(y)x-b \bigr\Vert _{2}^{2}+\lambda\mu P_{a}(x)-\mu \bigl\Vert F(y)x-F(y)y \bigr\Vert _{2}^{2}+ \Vert x-y \Vert _{2}^{2}. $$

It is clear that $C_{2}(x,x)=\mu C_{1}(x)$ for all $\mu>0$.

#### Theorem 1

*For any* $\lambda>0$ *and* $0<\mu<L_{\ast}^{-1}$ *with* $\Vert F(x^{\ast })x-F(x^{\ast})x^{\ast} \Vert _{2}^{2}\leq L_{\ast} \Vert x-x^{\ast} \Vert _{2}^{2}$. *If* $x^{\ast}$ *is the optimal solution of* $\min_{x\in\mathbb {R}^{n}}C_{1}(x)$, *then* $x^{\ast}$ *is also the optimal solution of* $\min_{x\in\mathbb{R}^{n}}C_{2}(x,x^{\ast})$, *that is*,

$$C_{2}\bigl(x^{\ast},x^{\ast}\bigr)\leq C_{2} \bigl(x,x^{\ast}\bigr) $$

*for any* $x\in\mathbb{R}^{n}$.

#### Proof

By the definition of $C_{2}(x, y)$, we have

$$\begin{aligned} C_{2}\bigl(x,x^{\ast}\bigr)&=\mu \bigl\Vert F \bigl(x^{\ast}\bigr)x-b \bigr\Vert _{2}^{2}+\lambda \mu P_{a}(x)-\mu \bigl\Vert F\bigl(x^{\ast}\bigr)x-F \bigl(x^{\ast}\bigr)x^{\ast} \bigr\Vert _{2}^{2}+ \bigl\Vert x-x^{\ast} \bigr\Vert _{2}^{2} \\ &\geq\mu \bigl\Vert F\bigl(x^{\ast}\bigr)x-b \bigr\Vert _{2}^{2}+\lambda\mu P_{a}(x) \\ &\geq \mu C_{1}\bigl(x^{\ast}\bigr) \\ &=C_{2}\bigl(x^{\ast},x^{\ast}\bigr). \end{aligned}$$

 □

#### Theorem 2

*For any* $\lambda>0$, $\mu>0$ *and solution* $x^{\ast}$ *of* $\min_{x\in\mathbb{R}^{n}}C_{1}(x)$, $\min_{x\in\mathbb {R}^{n}}C_{2}(x,x^{\ast})$ *is equivalent to*

21 $$ \min_{x\in\mathbb{R}^{n}} \bigl\{ \bigl\Vert x-B_{\mu}\bigl(x^{\ast}\bigr) \bigr\Vert _{2}^{2}+ \lambda\mu P_{a}(x) \bigr\} , $$

*where* $B_{\mu}(x^{\ast})=x^{\ast}+\mu F(x^{\ast})^{\top }(b-F(x^{\ast})x^{\ast})$.

#### Proof

By the definition, $C_{2}(x,y)$ can be rewritten as

$$\begin{aligned} C_{2}\bigl(x,x^{\ast}\bigr)={}& \bigl\Vert x- \bigl(x^{\ast}-\mu F\bigl(x^{\ast}\bigr)^{\top}F \bigl(x^{\ast }\bigr)x^{\ast}+\mu F\bigl(x^{\ast} \bigr)^{\top}b\bigr) \bigr\Vert _{2}^{2}+\lambda\mu P_{a}(x)+\mu \Vert b \Vert _{2}^{2}+ \bigl\Vert x^{\ast} \bigr\Vert _{2}^{2} \\ &{}-\mu \bigl\Vert F\bigl(x^{\ast}\bigr)x^{\ast} \bigr\Vert _{2}^{2}- \bigl\Vert x^{\ast}-\mu F \bigl(x^{\ast }\bigr)^{\top}F\bigl(x^{\ast} \bigr)x^{\ast}+\mu F\bigl(x^{\ast}\bigr)^{\top}b \bigr\Vert _{2}^{2} \\ ={}& \bigl\Vert x-B_{\mu}\bigl(x^{\ast}\bigr) \bigr\Vert _{2}^{2}+\lambda\mu P_{a}(x)+\mu \Vert b \Vert _{2}^{2}+ \bigl\Vert x^{\ast} \bigr\Vert _{2}^{2}-\mu \bigl\Vert F\bigl(x^{\ast} \bigr)x^{\ast} \bigr\Vert _{2}^{2}- \bigl\Vert B_{\mu}\bigl(x^{\ast}\bigr) \bigr\Vert _{2}^{2}, \end{aligned}$$

which implies that $\min_{x\in\mathbb{R}^{n}}C_{2}(x,x^{\ast})$ for any $\lambda>0$, $\mu>0$ is equivalent to

$$\min_{x\in\mathbb{R}^{n}} \bigl\{ \bigl\Vert x-B_{\mu} \bigl(x^{\ast}\bigr) \bigr\Vert _{2}^{2}+\lambda\mu P_{a}(x) \bigr\} . $$

 □

Combining Theorem 2, Theorem 1 and Lemma 1, the thresholding representation of $(QP_{a}^{\lambda})$ can be concluded by

22 $$ x^{\ast}=G_{a,\lambda\mu}\bigl(B_{\mu} \bigl(x^{\ast}\bigr)\bigr), $$

where the operator $G_{a,\lambda\mu}$ is defined in Definition 1 and obtained by replacing *λ* with *λμ*. With the thresholding representations (22), the IFTA for solving the regularization problem $(QP_{a}^{\lambda})$ can be naturally defined as

23 $$ x^{k+1}=G_{a, \lambda\mu}\bigl(B_{\mu} \bigl(x^{k}\bigr)\bigr),\quad k=0,1,2,\ldots, $$

where $B_{\mu}(x^{k})=x^{k}+\mu F(x^{k})^{\top}(b-F(x^{k})x^{k})$.

### Adjusting the values for the regularization parameter $\lambda>0$

In this subsection, the cross-validation method (see [9, 10, 12]) is accepted to automatically adjust the value for the regularization parameter $\lambda>0$. In other words, when some prior information is known for a regularization problem, this selection is more reasonable and intelligent. Suppose that the vector $x^{\ast}$ of sparsity *r* is the optimal solution of the regularization problem $(QP_{a}^{\lambda})$, and without loss of generality, set

$$\bigl\vert B_{\mu}\bigl(x^{\ast}\bigr) \bigr\vert _{1}\geq \bigl\vert B_{\mu}\bigl(x^{\ast}\bigr) \bigr\vert _{2}\geq\cdots\geq \bigl\vert (B_{\mu} \bigl(x^{\ast}\bigr) \bigr\vert _{r}\geq \bigl\vert (B_{\mu}\bigl(x^{\ast}\bigr) \bigr\vert _{r+1}\geq \cdots \geq \bigl\vert (B_{\mu}\bigl(x^{\ast}\bigr) \bigr\vert _{n}\geq0. $$

Then it follows from (14) that

$$\begin{aligned} &\bigl\vert B_{\mu}\bigl(x^{\ast}\bigr) \bigr\vert _{i}>t_{a,\lambda\mu}^{\ast}\quad\Leftrightarrow\quad i\in\{1,2,\ldots,r \}, \\ &\bigl\vert B_{\mu}\bigl(x^{\ast}\bigr) \bigr\vert _{i}\leq t_{a,\lambda\mu}^{\ast}\quad\Leftrightarrow\quad i\in\{r+1,r+2, \ldots,n\}, \end{aligned}$$

where $t_{a,\lambda\mu}^{\ast}$ is obtained by replacing *λ* with *λμ* in $t_{a,\lambda}^{\ast}$.

By $t_{a,\lambda\mu}^{2}\leq t_{a,\lambda\mu}^{1}$, we have

24 $$ \textstyle\begin{cases} \vert B_{\mu}(x^{\ast}) \vert _{r}\geq t_{a,\lambda\mu}^{\ast}\geq t_{a,\lambda\mu}^{2}=\sqrt{\lambda\mu}-\frac{1}{2a}; \\ \vert B_{\mu}(x^{\ast}) \vert _{r+1}< t_{a,\lambda\mu}^{\ast}\leq t_{a,\lambda \mu}^{1}=\frac{\lambda\mu}{2}a. \end{cases} $$

It follows that

25 $$ \frac{2 \vert B_{\mu}(x^{\ast}) \vert _{r+1}}{a\mu}\leq\lambda\leq\frac {(2a \vert B_{\mu}(x^{\ast}) \vert _{r}+1)^{2}}{4a^{2}\mu}. $$

From (25), we obtain

$$\lambda\in \biggl[\frac{2 \vert B_{\mu}(x^{\ast}) \vert _{r+1}}{a\mu}, \frac {(2a \vert B_{\mu}(x^{\ast}) \vert _{r}+1)^{2}}{4a^{2}\mu} \biggr]. $$

We denote by $\lambda_{1}$ and $\lambda_{2}$ the left and the right of the above interval, respectively:

$$\lambda_{1}=\frac{2 \vert B_{\mu}(x^{\ast}) \vert _{r+1}}{a\mu}\quad \text{and}\quad \lambda_{2}= \frac{(2a \vert B_{\mu}(x^{\ast }) \vert _{r}+1)^{2}}{4a^{2}\mu}. $$

A choice of *λ* is

$$\lambda=\textstyle\begin{cases} \lambda_{1} & \text{if } \lambda_{1}\leq\frac{1}{a^{2}\mu }; \\ \lambda_{2} & \text{if } \lambda_{1}>\frac{1}{a^{2}\mu}. \end{cases} $$

Since $x^{\ast}$ is unknown, and $x^{k}$ is the best available approximation to $x^{\ast}$, so we can take

26 $$ \lambda=\textstyle\begin{cases} \lambda_{1,k}=\frac{2 \vert B_{\mu}(x^{k}) \vert _{r+1}}{a\mu} & \text{if } \lambda_{1,k}\leq\frac{1}{a^{2}\mu}; \\ \lambda_{2,k}=\frac{(2a \vert B_{\mu}(x^{k}) \vert _{r}+1)^{2}}{4a^{2}\mu} & \text{if } \lambda_{1,k}>\frac{1}{a^{2}\mu}, \end{cases} $$

in the *k*th iteration. That is, (26) can be used to automatically adjust the value of the regularization parameter $\lambda >0$ during iteration.

#### Remark 1

Notice that (26) is valid for any $\mu>0$ satisfying $0<\mu \leq \Vert F(x_{k}) \Vert _{2}^{-2}$. In general, we can take $\mu=\mu _{k}=\frac{1-\epsilon}{ \Vert F(x_{k}) \Vert _{2}^{2}}$ with any small $\epsilon\in(0,1)$ below. Especially, the threshold value is $t_{a,\lambda\mu}^{\ast}=\frac{\lambda\mu}{2}a$ when $\lambda=\lambda_{1,k}$, and $t_{a,\lambda\mu}^{\ast}=\sqrt{\lambda\mu}-\frac{1}{2a}$ when $\lambda=\lambda_{2,k}$.

### Iterative fraction thresholding algorithm (IFTA)

Based on the thresholding representation (23) and the analyses given in Sect. 3.2, the proposed iterative fraction thresholding algorithm (IFTA) for regularization problem $(QP_{a}^{\lambda})$ can be naturally described in Algorithm 1.

**Algorithm 1:**
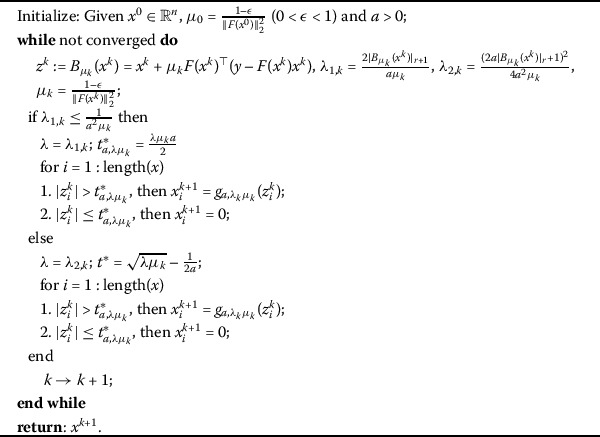
Iterative fraction thresholding algorithm (IFTA)

#### Remark 2

The convergence of IFTA is not proved theoretically in this paper, and this is our future work.

## Numerical experiments

In the section, we carry out a series of simulations to demonstrate the performance of IFTA, and compare them with those obtained with some state-of-art methods (iterative soft thresholding algorithm (ISTA) [6, 7]), iterative hard thresholding algorithm (IHTA) [6, 7]. In our numerical experiments, we set

27 $$ F(x)=A_{1}+\eta f\bigl( \Vert x-x_{0} \Vert _{2}\bigr) A_{2}, $$

where $A_{1}\in\mathbb{R}^{100\times400}$ is a fixed Gaussian random matrix, $x_{0}\in\mathbb{R}^{400}$ is a reference vector, $f:[0,\infty)\rightarrow\mathbb{R}$ is a positive and smooth Lipschitz continuous function with $f(t)=\ln(t+1)$, *η* is a sufficiently small scaling factor (we set $\eta=0.003$), and $A_{2}\in \mathbb{R}^{30\times100}$ is a fixed matrix with every entry equals 1. Then the form of nonlinearity considered in (27) is a quasi-linear, and the more detailed accounts of the setting in the form of (27) can be found in [6, 7]. By randomly generating such sufficiently sparse vectors $x_{0}$ (choosing the nonzero locations uniformly over the support in random, and their values from $N(0,1)$), we generate vectors *b*. In this way, we know the sparsest solution to $F(x_{0})x_{0} = b$, and we are able to compare this with algorithmic results. The stopping criterion is usually as follows:

$$\frac{ \Vert x_{k}-x_{k-1} \Vert _{2}}{ \Vert x_{k} \Vert _{2}}\leq\mathrm{Tol}, $$

where $x_{k}$ and $x_{k-1}$ are numerical results from two continuous iterative steps and Tol is a given small number. The success is measured by computing

$$\text{relative error}=\frac{ \Vert x^{\ast}-x_{0} \Vert _{2}}{ \Vert x_{0} \Vert _{2}}\leq\mathrm{Re}, $$

where $x^{\ast}$ is the numerical results generated by IFTA, and Re is also a given small number. In all of our experiments, we set $\mathrm{Tol}=10^{-8}$ to indicate the stopping criterion, and set $\mathrm{Re}=10^{-4}$ to indicate a perfect recovery of the original sparse vector $x_{0}$.

**Figure 8:**
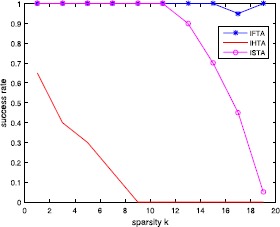
Success rate of three algorithms in the recovery of a sparse signal with different cardinality. In this experiment, we repeatedly perform 30 tests and present average results and take $a=2.5$

Figure 8 shows the success rate of three algorithms in the recovery of a sparse signal with different cardinality. In this experiment, we repeatedly perform 30 tests and present average results and take $a=2.5$.

**Figure 9:**
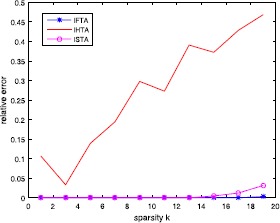
Relative error between the solution $x^{\ast}$ and the given signal $x_{0}$. In this experiment, we repeatedly perform 30 tests and present average results and take $a=2.5$

Figure 9 shows the relative error between the solution $x^{\ast}$ and the given signal $x_{0}$. In this experiment, we repeatedly perform 30 tests and present average results and take $a=2.5$.

The graphs presented in Fig. 8 and Fig. 9 show the performance of the ISTA, IHTA and IFTA in recovering the true (sparsest) signals. From Fig. 8, we can see that IFTA performs best, and IST algorithm the second. From Fig. 9, we see that the IFTA has the smallest relative error value with sparsity growing.

## Conclusion

In this paper, we take the fraction function as the substitution for $\ell_{0}$-norm in quasi-linear compressed sensing. An iterative fraction thresholding algorithm is proposed to solve the regularization problem $(QP_{a}^{\lambda})$ for all $a>0$. With the change of parameter $a>0$, our algorithm could get a promising result, which is one of the advantages for our algorithm compared with some state-of-art algorithms. We also provide a series of experiments to assess performance of our algorithm and the experiment results have illustrated that our algorithms is able to address the sparse signal recovery problems in nonlinear systems. Compared with ISTA and IHTA, IFTA performs best in sparse signal recovery and has the smallest relative error value with sparsity growing. However, the convergence of our algorithm is not proved theoretically in this paper, and it is our future work.
